# Everybody's Page

**Published:** 1889-08-03

**Authors:** 


### Everybody's Page.

The Paris *Journal de Médécine* has begun to publish a list of persons who have died from hydrophobia after being treated by Pasteur.

Myopia is found in American school-children in the proportion of about five per cent., and always in the higher classes of the school.

Ordinary freckles may in exceptional cases develop into the disease called xero-derma pigmentosum, in which the freckles coalesce, and become inflamed and ulcerous.

The statistics presented at the last meeting of the Metropolitan Asylums Board show a slight increase of fever during the last fortnight, 237 persons having been admitted to the hospitals under the control of the Board, as against 204 in the previous fortnight.

Our good friends, the Socialists, who are always championing the "slaves" in one trade or another, ought to extend their sympathy to the police. These unfortunate men, when a royal wedding or a Whitechapel murder causes an extra pressure on their services, have to work fourteen hours a day for seven days a week, and without any extra pay. Surely this deserves the attention of the *Commonweal.*

The negroes of the southern state of America have some curious customs with regard to their treatment of the dead, one of which is not without interest to the medical profession. They lay on the grave all the medicine bottles, whether empty or partly filled, that have been used by the dead person during his last illness. It is not stated whether or not this is meant as a compliment to the doctor or a reproach to him; but we should think he doesn't quite like these visible reminders of his failure to save his patient's life.

Dr. Arthur Jamison records an interesting case of the removal of superfluous hair. The patient, whom he began treating when she was three months' old, was a girl, the right-half of whose face was covered with long hair; in fact, Dr. Jamison says that on that side she presented the appearance of a skye terrier. After thinking over various remedies he tried rubbing in sodium ethylate, and by this means succeeded in removing all the superfluous hair, though more than one application was necessary to conquer some of the most stubborn of the hair follicles; and the child was six years' old before the doctor considered that the cure was complete. One advantage of the ethylate is that it does not disfigure the skin itself as many depilatories do; but of course it must be employed with the greatest care and discretion.

The mental effects of solitary confinement on the prisoner have been discussed recently in France. Dr. de Pietra Santa, who is a well-known authority on hygiene, has studied the matter at the prison at Mazas, and has come to the conclusion that this form of imprisonment develops a tendency to melancholia, with an inclination for suicide, even where there is no such predisposition before the imprisonment begins. During the last fourteen years, 32 prisoners have committed suicide at Mazas, though facilities for self-destruction are conspicuously wanting there. Dr. Voisin, on the other hand, whose experience is of Belgian prisons, thinks that in time the prisoner looks upon his cell as his home. But in Belgium absolute loneliness is not so rigidly enforced as in France, and the prisoner has visits not only from his gaoler, but from teachers, technical and others, whose instructions, little as he may desire them, keep him from going melancholy mad.

During the last 80 years eight million people have died of phthisis in France.

Owing to its astringent action, an infusion of kola powder is often suitable for people who find tea or coffee disagreeable in their after-effects.

The reason of the greater mortality of male children than of female is supposed to be the greater demands on the system caused by their more rapid rate of growth.

Dr. A. V. Meigs, the well-known physician of Philadelphia, recommends the use of opium in combination with ipecachuana as a remedy in spasmodic croup. The dose should be small—not more than two drops of laudanum to ten of ipecac syrup; but they may be repeated hourly without danger.

Consumption is rare in childhood, but increases rapidly after the age of fifteen, and is most common between the ages of twenty-five and thirty. Those who escape it till the latter age are less and less prone to it as the years advance, and may escape it entirely even though they have a hereditary predisposition to it.

The importance of light in relation to development has been shown by an interesting experiment in the growth of tadpoles. A scientist brought up a dozen tadpoles in boxes from which light was excluded, although every other condition necessary to life was present. Of the twelve, only two grew into frogs, and that at a much later period than usual. The remaining ten increased in size, but never grew out of the tadpole estate.

Dr. Alexander James, of Edinburgh, has recently published an interesting volume on phthisis. He accepts the bacillus as the primary factors in consumption, but he does not ride microbomania to death. He admits that the bacillus may remain inactive for years if the "soil" in which it is cast is unsuitable for its development; if, in other words, the general nutrition of the body be satisfactory. In practical language this means: Take care of your general health, and even with a hereditary predisposition to consumption you may never be attacked by it.

Some over-careful parents are so anxious that their children should not grow up lazy that they do not allow them as much sleep as they require. Growth requires sleep as much as exercise. It is during sleep that the system repairs the losses caused by the exertions of the day, and growth is an accelerated form of the process of repair. To facilitate healthy growth abundance of sleep is required. During the first year of life, in which an infant's weight at birth is trebled, a great part of its time is spent in sleep, and in modified proportions this need of rest goes on through the whole period of growth.

The English are supposed to love fresh air, but a believer in free ventilation has hard measure dealt out to him by most of those who come in contact with him. If he travels by rail the ladies in the carriage are afraid of draught, or dust, or something, and insist on keeping the windows closed. If he opens a window, except in the height of summer, his wife or servants shut it directly; and the builder is almost certain to have been in the plot to asphyxiate him. An admirable chimney has been secured in a certain block of "model" flats, where the rooms which have fireplaces are also provided with ventilators, while those which are left without chimneys are also left without any means of permitting the ingress of air except a window that will not open more than three inches.

